# Snake Bites

**Published:** 1882-11

**Authors:** 


### METHOD OF EMPLOYING PERMANGANATE OF POTASS AS ANTIDOTE TO THE VENOM OF SERPENTS.

[From the *Journal d'Hygiéne.* Translated from the French by the Editor of HALL's JOURNAL OF HEALTH.]

M. DE LACERDA, of Rio Janeiro, has had the kindness to condense in an accurate note, which has received the greatest publicity, the precautions necessary in the application of his remedy for bites and stings of serpents, on men as well as animals.

The permanganate of potass ought not to be introduced by the digestive passages, but applied *in situ*, that is to say, injected into the cellular tissue or into the veins, according to whether the bite is superficial or deep.

The successive march of the symptoms indicates perfectly if the venom of the serpent has limited its action to the cellular tissue, or if it has entered the veins. In the first case local symptoms predominate, with slight appearance of morbid symptoms ; in the second, the general phenomena appear with as great rapidity as intensity and gravity.

For hypodermic injections, after a ligature is applied to the member above the wound, introduce the needle, as near as possible in the track of the bite, and push gradually the piston of the syringe, filled with a solution of permanganate of potass 1 to 100. Operate in preference on the most superficial veins. According to the gravity of the case, two to three syringefuls of the liquid may be injected.

At the same time that the injections are being applied, it is well to administer to the patient exciting and tonic drinks, in order to combat symptoms of prostration.

It has been suggested that this remedy might be employed effectively in bites of mad dogs, either as a cautery or injection, hypodermically, or both combined.

---

OLD boot-tops lined make excellent iron-holders.

IT IS SAID that "about the year 1796 there was offered as a gift to a Lutheran Church in New York city six acres of land, near Canal street and Broadway; and it was declined, as not being worth fencing."

